# Exosomes in liver fibrosis: The role of modulating hepatic stellate cells and immune cells, and prospects for clinical applications

**DOI:** 10.3389/fimmu.2023.1133297

**Published:** 2023-03-20

**Authors:** Yufei Liu, Yuhong Zheng, Yang Yang, Ke Liu, Jianying Wu, Peiyang Gao, Chuantao Zhang

**Affiliations:** ^1^ Department of Respiratory Medicine, Hospital of Chengdu University of Traditional Chinese Medicine, Chengdu, China; ^2^ Department of Digestive Medicine, Hospital of Chengdu University of Traditional Chinese Medicine, Chengdu, China; ^3^ Department of Critical Care Medicine, Hospital of Chengdu University of Traditional Chinese Medicine, Chengdu, China

**Keywords:** exosome, liver fibrosis, immune cell, hepatic stellate cell, clinical application

## Abstract

Liver fibrosis is a global health problem caused by chronic liver injury resulting from various factors. Hepatic stellate cells (HSCs) have been found to play a major role in liver fibrosis, and pathological stimuli lead to their transdifferentiation into myofibroblasts. Complex multidirectional interactions between HSCs, immune cells, and cytokines are also critical for the progression of liver fibrosis. Despite the advances in treatments for liver fibrosis, they do not meet the current medical needs. Exosomes are extracellular vesicles of 30-150 nm in diameter and are capable of intercellular transport of molecules such as lipids, proteins and nucleic acids. As an essential mediator of intercellular communication, exosomes are involved in the physiological and pathological processes of many diseases. In liver fibrosis, exosomes are involved in the pathogenesis mainly by regulating the activation of HSCs and the interaction between HSCs and immune cells. Serum-derived exosomes are promising biomarkers of liver fibrosis. Exosomes also have promising therapeutic potential in liver fibrosis. Exosomes derived from mesenchymal stem cells and other cells exhibit anti-liver fibrosis effects. Moreover, exosomes may serve as potential therapeutic targets for liver fibrosis and hold promise in becoming drug carriers for liver fibrosis treatment.

## 1 Introduction

Liver fibrosis (LF) is caused by chronic liver injury from various causes, such as viral infections, alcohol consumption, non-alcoholic fatty liver disease (NAFLD), cholestatic liver disease, and autoimmune hepatitis. It is characteristic of extracellular matrix (ECM) deposition in the liver (1). The pathogenesis of liver fibrosis is complex, involving the activation of hepatic stellate cells, the role of hepatic macrophages, the role of cytokines (2). It is well known that chronic liver disease is a worldwide health dilemma (3). Among chronic liver diseases, LF has a high prevalence and largely affects the quality of life and disease prognosis, making it a health challenge (4, 5). Continued progression of LF can lead to cirrhosis, which causes approximately 1 million deaths worldwide each year (3). Moreover, about 80% to 90% of patients with hepatocellular carcinoma have a history of LF (6). Therefore, early diagnosis and effective treatment of LF are essential in reducing the global health burden.

Liver biopsy remains the gold standard for the diagnosis and prognosis of LF but has some limitations, such as invasiveness, economic cost, complications, and sampling errors (7). Several non-invasive diagnostic measures of LF are available, such as liver stiffness measurement (LSM), aspartate aminotransferase to platelet ratio index (APRI), and fibrosis-4 index (FIB-4) (8). However, the diagnostic effectiveness of these measures is influenced by the disease state and in some cases has only moderate diagnostic performance (9). Therefore, non-invasive diagnostic measures with high efficacy are still needed to assist clinicians in the early diagnosis of LF. In recent years, a large number of researches have been invested in the development of anti-LF drugs. However, few of them have been able to exert good anti-LF effects in clinical trials. There is still a lack of effective drugs to treat or even reverse LF (2, 5).

Exosomes are a type of extracellular vesicles (EVs) secreted by most cells, capable of carrying various cellular components and playing an essential role in intercellular communication (10). Exosomes can participate in the development of multiple diseases, such as cardiovascular diseases, metabolic diseases, and immune diseases. An increasing number of studies are devoted to the utility of exosomes in diagnosing and treating various diseases (10, 11). In this review, we summarize the role of exosomes in LF pathogenesis, particularly in regulating hepatic stellate cells (HSCs) and immune cells in LF. Furthermore, we discuss the diagnostic and therapeutic potential of exosomes in LF.

## 2 Exosome

Exosomes are nanoscale lipid bilayer vesicles secreted by the majority of cells, ranging from 30-150 nm in diameter (12). In the 1980s, exosomes were first discovered in reticulocytes and were named “Exosome” by Johnstone et al (13–16). However, for some time afterwards, exosomes were considered a way that cells excrete waste products and did not receive much attention (17, 18). In the late 1990s, exosomes produced by B-lymphocytes and dendritic cells were found to have antigen-presenting properties, which led to an interest in exosomes in the field of immunology (19, 20). In 2007, Valadi et al. discovered that exosomes could carry mRNA and microRNA(miRNA) for intercellular exchange of genetic material, leading to a new level of research on exosomes (21). With the extensive research conducted on exosomes, they are nowadays considered to play an important role in intercellular communication. Exosomes are involved in the development of various diseases by transporting the corresponding lipids, proteins and nucleic acids, as well as having high clinical applications (11).

### 2.1 Composition of exosome

**Figure 1:**
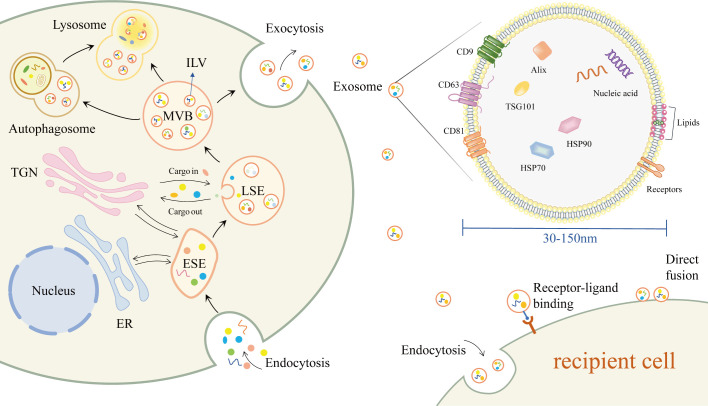
Biogenesis, composition, secretion and uptake of exosomes.

Exosomes have a lipid bilayer structure and contain a variety of proteins, lipids and nucleic acids (**Figure 1**) (22). Some proteins are commonly found in various exosomes, such as tetraspanins (e.g. CD9, CD63, CD81), heat shock proteins (e.g. HSP70, HSP90), endosomal sorting complex required for transport (ESCRT)-related proteins (e.g. Alix, TSG101) (22, 23). In addition, due to cellular specificity, different exosomes may have specific proteins, such as MHC-II (24). The lipids of exosomes contain sphingolipids, cholesterol, phosphatidylcholine, phosphatidylserine, etc (25). Various nucleic acids are present in exosomes, including DNA, mRNA, miRNA and other non-coding RNAs(ncRNA) (26).

### 2.2 Biogenesis, secretion and uptake of exosome

The production of exosomes begins with the double invagination of the plasma membrane, where extracellular components and cell membrane proteins enter the cell with the invagination of the plasma membrane to form early-sorting endosome(ESE) (11). The endoplasmic reticulum (ER) and the trans-Golgi network (TGN) can also be involved in the formation of ESE (11, 27, 28). The ESE then matures into late-sorting endosome (LSE) and eventually into multivesicular body (MVB). The MVB is enriched with intraluminal vesicles (ILVs), which are produced by the inward budding of the endosomal membrane (10). MVBs fuse with the plasma membrane to release the contained ILVs outside the cell as exosomes. Other MVBs are degraded by direct fusion with lysosomes or by fusion with autophagosomes and subsequent degradation in lysosomes (22).

After delivery to the recipient cell, exosomes can be taken up through different pathways. Exosomes can fuse directly with the plasma membrane, or enter the recipient cell *via* endocytosis (29). Exosomes can also induce downstream signaling cascade responses in recipient cells *via* receptor-ligand interactions (22, 30).

## 3 Exosomes in LF pathogenesis

LF is a response to the multiple etiologies of chronic liver injury (1). Liver injury leads to hepatocyte damage with immune cell infiltration. Quiescent HSCs (qHSCs) are thus activated and transformed into myofibroblasts, which are involved in tissue repair under normal conditions. In short-term liver injury, the body’s pro-fibrotic and anti-fibrotic mechanisms are in balance, and LF is not likely to occur. However, when the chronic liver injury occurs, hepatocytes become necrotic and apoptotic, and damaged hepatocytes release damage-associated molecular patterns (DAMPs). DAMPs directly activate the fibrotic phenotype of HSCs, producing a large amount of ECM with type I and type III collagen and fibronectin as the main components. Activated HSCs secrete cytokines such as transforming growth factor β1 (TGF-β1), platelet-derived growth factor (PDGF) and connective tissue growth factor (CTGF). Autocrine secretion of activated HSC (aHSC) further constantly activates qHSC (2). DAMPs also induce the recruitment and activation of immune cells such as macrophages. These immune cells promote HSC activation and myofibroblast production by secreting pro-inflammatory and pro-fibrotic factors (5, 31).

**Figure 2:**
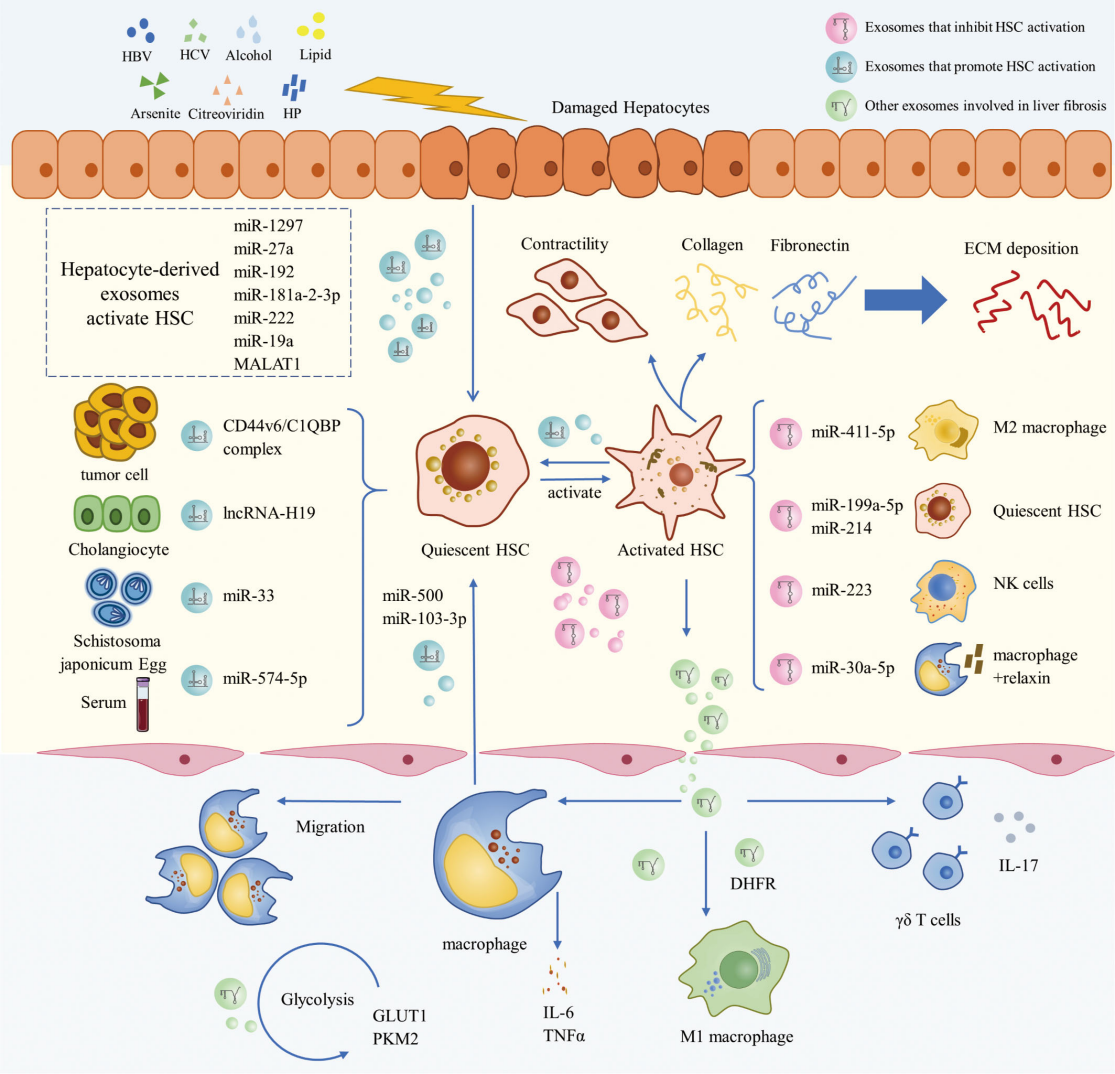
The role of exosomes in the pathogenesis of LF.

Complex multidirectional interactions exist between HSCs, immune cells, and cytokines. HSCs are continuously activated and proliferated by paracrine and autocrine. And the secretion of abundant pro-fibrotic cytokines and excessive ECM production lead to the disruption of the balance of pro-fibrotic/anti-fibrotic mechanisms, eventually leading to the formation of LF (2, 5). As the study of exosomes and LF has gradually advanced, exosomes have been found to play a non-negligible role in the biological processes of HSC activation and HSC-immune cell interaction in LF (**Figure 2**).

### 3.1 Exosomes are involved in the activation of HSCs in LF

In the normal liver, HSCs are quiescent and function in vitamin A metabolism and storage, fat storage, and matrix metalloproteinase synthesis. After the liver injury, HSCs are activated and transdifferentiated from a quiescent phenotype to a proliferative and contractile myofibroblast phenotype. As the major source of myofibroblasts, HSC activation is critical for the progression of LF (32). HSC activation consists of two main phases, initiation and perpetuation. The initiation phase is an early change in gene expression in HSC caused by multiple extracellular signals, mainly paracrine stimuli. It is mainly triggered by products of damaged hepatocytes, signals from Kupffer cells, reactive oxygen species and lipid peroxide exposure, and extracellular matrix changes (33). Perpetuation encompasses the maintenance of the HSC activation phenotype and the development of fibrosis. Perpetuation involves paracrine and autocrine, and these constant stimuli lead to LF by enhancing HSC proliferation, contractility, fibrosis, matrix degradation, and pro-inflammatory signaling (33, 34).

Damage to hepatocytes, resulting in the activation of HSC, is the initial step of LF formation (2). Exosomes derived from hepatocytes can transfer biological messages to promote HSC activation, thus contributing to the progression of LF. Fatty liver disease is a significant contributor to the development of LF (1). Under the stress conditions of fatty liver disease, hepatocyte-derived exosomes can induce the activation of quiescent HSCs (qHSCs) to a myofibroblast phenotype (35). Lipotoxic hepatocyte-derived exosomal miR-1297 could target the PTEN/PI3K/AKT signaling pathway, which in turn promotes the activation and proliferation of HSC and accelerates the progression of LF (36). Activated HSCs are also the recipient cells of lipotoxic hepatocyte-derived exosomes (37). Xin Luo et al. found that lipotoxic hepatocyte-derived exosomal miR-27a could target protein kinase 1 in aHSC and thereby inhibit mitochondrial autophagy. They demonstrated that lipotoxic hepatocyte-derived exosomal miR-27a advances the progression of LF in metabolism-associated fatty liver disease (MAFLD) by inhibiting autophagy in aHSC and enhancing HSC proliferation and activation (37). Viral infection is one of the leading causes of LF, and exosomes play an important role in this process (38). During hepatitis B virus (HBV) infection, exosomes derived from HBV-infected hepatocytes promote HSC activation and LF in mice. Among them, the exosomal miR-222 could enhance HSC activation by inhibiting transferrin receptor (TFRC)-induced HSC iron death (39). Exosomes from hepatitis C virus (HCV)-infected hepatocytes can also induce HSC activation. Exosomal miR-19a can be delivered from HCV-infected hepatocytes to HSC and activate HSC through STAT3/TGF-β1/Smad3 signaling pathway (40). Similarly, exosomal miR-192 released from HCV-replicating hepatocytes could be involved in HCV-induced LF by promoting HSC activation and transdifferentiation (41). In recent years, Helicobacter pylori infection has been suggested to play a role in several liver diseases. Zahmatkesh et al. demonstrated that Exosomes derived from hepatocytes infected with helicobacter pylori outer membrane vesicle can lead to overexpression of HSC activation markers and fibrosis markers, suggesting a possible role in HSC activation and LF progression (42). Dai et al. provided new insight into the mechanism of arsenic toxicity-induced LF. They observed that hepatocyte-derived exosomal metastasis associated in lung denocarcinoma transcript 1(MALAT1) could induce HSC activation in the arsenite-induced LF model, which is associated with the regulation of collagen type I alpha2 (COL1A2) by exosomal MALAT1 through miRNA-26b (43). In addition, Dong et al. found that hepatocyte-derived exosomes offer new clues to the mechanism of citreoviridin hepatotoxicity and LF. They indicated that exosomal miR-181a-2-3p derived from citreoviridin-treated hepatocytes could activate HSC. It is associated with the induction of mitochondrial calcium overload by exosomal miR-181a-2-3p through inhibition of mitochondrial calcium uptake 1 expression (44).

Exosomes derived from various cells other than hepatocytes have also been reported to promote the activation of HSC and LF. It is demonstrated that exosomes may be involved in tumor-associated LF through the activation of HSC. Feng et al. suggested that fluid shear stress-induced exosomes from liver cancer cells may promote HSC activation and proliferation, which is the main source of activation of cancer-associated fibroblasts (45). In addition, Xie et al. showed that exosomes could provide a hepatic fibrosis microenvironment for driving pancreatic cancer liver metastasis. They found that pancreatic ductal adenocarcinoma-derived exosomal CD44v6/C1QBP complex can be delivered to the plasma membrane of HSC, leading to phosphorylation of insulin-like growth factor 1 signaling molecules, which results in HSC activation and LF (46). There is a direct connection between cholangiocytes and HSCs in the pathogenesis of cholestatic LF. Exosomes derived from cholangiocytes are found to be preferentially taken up by HSCs compared to other hepatic cells. Notably, the cholangiocyte-derived exosomal lncRNA-H19 plays a vital role in the progression of cholestatic LF by promoting HSC activation and transdifferentiation (47). LF is the main pathological feature of schistosomiasis japonica. Wang et al. found that miRNA-33 carried by Schistosoma japonicum egg-derived exosomes could activate HSC and promote schistosomiasis LF in mice (48). It is widely accepted that the crosstalk between HSCs and macrophages plays an important role in LF. Exosomes released from macrophages have been reported to activate HSC. Chen et al. found that exosomal miR-500 released from lipopolysaccharide (LPS)-treated macrophages could be taken up by HSCs. Moreover, exosomal miR-500 could promote HSC activation and proliferation by targeting MFN2 to inhibit TGF-β/Smad, which in turn accelerates LF (49). In another study, Chen et al. found that exosomal miR-103-3p released from LPS-treated macrophages could also promote the activation and proliferation of HSC and thus participate in LF, which may be associated with miR-103-3p targeting Krüppel-like factor 4 (50). In addition, it has been reported that circulating exosomes may carry miR-574-5p to HSCs during LF, thereby stimulating HSC activation and collagen synthesis. However, the underlying mechanism remains to be elucidated (51).

Notably, it is reported that exosomes released from aHSC could be delivered to qHSC and promote its activation (52). Fang et al. found that apoptosis signal-regulated kinase 1-mediated endoplasmic reticulum stress may lead to the release of exosomes from angiotensin II-activated HSCs. Furthermore, these exosomes from aHSC subsequently activate qHSC and thus participate in the progression of LF (53). Additionally, exosomes released from qHSC have been found to be able to block fibrotic signaling in aHSC. Exosomal miR-199a-5p released by qHSC can be transferred to aHSC to inhibit the expression of CTGF (54). Similarly, exosomal miRNA-214 can be transferred from qHSCs to neighboring aHSCs and hepatocytes, resulting in the inhibition of CTGF and its downstream targets (55). During LF, the decreased expression of these exosomal miRNAs can lead to an increase the expression of CTGF (54, 55).

In conclusion, exosomes have been demonstrated to be of great importance in the activation of HSC, thus aiding in the comprehension of the mechanisms of various chronic liver diseases and LF. The progression of LF is a critical factor influencing the prognosis of chronic liver diseases (2). The research results on exosome-mediated activation of HSC have enriched our understanding of the initiation and progression of LF, and are highly valuable in the clinical application of preventing the development of LF and improving the prognosis of chronic liver diseases.

### 3.2 Exosomes are involved in the interaction between HSCs and immune cells in LF

Interaction between HSCs and immune cells, such as macrophages, have a significant role in the development and progression of LF (31). As the mediator of intercellular communication, exosomes are reported to regulate the interactions between HSC and immune cells in LF.

In LF, immune cells are the primary source of profibrotic signals, and HSCs regulate these cells by releasing a range of cytokines and chemokines (31). Exosomes have been demonstrated to be involved in the regulation of immune cells by HSCs. HSC-derived exosomes have been reported to stimulate the release of cytokines such as IL-6 and TNFα from macrophages and stimulate macrophage migration, thereby modulating the macrophage inflammatory response and promoting LF (56, 57). During the development of LF, exosomes secreted by aHSC can further promote M1 polarization in macrophages, and the exosomal dihydrofolate reductase(DHFR) may play an important role in this process (58). Wan et al. found that exosomes released by aHSC could induce glycolysis in hepatic non-parenchymal cells such as Kupffer cells. They suggested that the glycolysis-related proteins Glucose transporter type 1(GLUT1) and Pyruvate kinase isozyme typeM2(PKM2) delivered by exosomes could affect the metabolic switch among hepatic non-parenchymal cells, which could be involved in LF (59). In addition, hepatic exosomes could mediate the activation of toll-like receptor 3 in HSCs, thereby stimulating γδ T cell production of IL-17 and exacerbating LF (60).

Studies have demonstrated that during the progression of LF, immune cells can regulate the activity of HSCs through exosomes derived from these cells, primarily modulating HSC activation. Macrophage-derived exosomal miRNAs such as miR-500 and miR-103-3p have been demonstrated to activate HSC, thereby promoting LF (49, 50). Wan et al. found that miR-411-5p delivered by exosomes of M2 macrophages could inhibit HSC activation in a nonalcoholic steatohepatitis model, which was associated with the downregulation of Calmodulin-Regulated Spectrin-Associated Protein by miR-411-5p (61). Another study found that after binding to relaxin, hepatic macrophages could mediate the inactivation of aHSC *via* miR-30a-5p in their released exosomes (62). In addition, exosomal miR-223 derived from natural killer cells was reported to target ATG7 to inhibit autophagy, thereby suppressing TGF-β1-induced HSC activation (63).

Exosomes have been demonstrated to play a critical role in the regulation of the interaction between HSCs and immune cells in LF. A thorough investigation of the underlying molecular mechanisms by which exosomes regulate HSCs and immune cells may provide a useful approach for disease progression monitoring and potential therapeutic strategies in the future.

## 4 Clinical application of exosomes in LF

### 4.1 Diagnostic potential of exosomes in LF

Accurate and timely diagnosis of LF is essential for effective disease management and improved prognosis. Exosomes are distributed in various biological fluids, such as blood, serum, urine, saliva, amniotic fluid, etc (22). The progression of the diseases could be tracked by detecting exosomes in body fluids (11). In recent years, the potential of exosomes as biomarkers in the diagnosis, staging, and prognosis of various diseases has attracted much attention (23). The detection of exosomes from patients’ blood samples may provide a less invasive way to diagnose LF (**Table 1**).

**Table 1:** Biomarker potential of exosomes in LF.

Exosome – Cargos	Expression in LF (vs. Controls)	Source	Clinical Implication	References
miR-92a-3p	Upregulated	Serum	Potential non-invasive biomarker of significant LF in chronic hepatitis B.	(64)
miR-146a-5p	Upregulated			
miR-122	Downregulated	Serum	Potential diagnostic biomarker for LF associated with chronic liver disease of non-viral etiology with the ability to identify advanced LF	(65)
miR-500	Upregulated	Serum	Potential diagnostic biomarker for advanced LF	(49)
miR-103-3p	Upregulated	Serum	Potential diagnostic biomarker for advanced LF	(50)
miR-27a	Upregulated	Serum	Potential diagnostic biomarker for MAFLD-associated LF	(37)
miR-574-5p	Upregulated	Serum	Potential diagnostic biomarker for LF	(51)
miR-155	Upregulated	Plasma	Potential diagnostic biomarker for LF; Promising prognostic biomarker for patients with LF undergoing liver transplantation	(66)
circDIDO1	Downregulated	Serum	Potential diagnostic biomarkers of LF in liver failure	(67)
lncRNA-H19	Upregulated	Serum	Potential diagnostic biomarker for cholestatic LF in biliary atresia	(68)
lncRNA-MALAT1	Upregulated	Serum	Potential diagnostic biomarker of LF in arsenicosis	(43)
CD44	Upregulated	Serum	Potential diagnostic biomarkers of LF in congestive hepatopathy	(69)

LF, Liver fibrosis; MAFLD, metabolism-associated fatty liver disease; CD44, cluster of differentiation of 44.

Exosomal miRNAs have been demonstrated to be involved in HSC activation, immune response, and other biological processes associated with LF. Furthermore, patients with LF exhibit altered levels of exosomal miRNAs, making them attractive candidates for diagnosing LF. In chronic hepatitis B, serum exosomes containing miR-92a-3p and miR-146a-5p have been identified as promising biomarkers for assessing LF. These biomarkers can be used to monitor the progression of LF and distinguish between the disease’s early and advanced stages (64). In chronic liver diseases of non-viral etiology, serum exosomal miR-122 has been established as a promising diagnostic tool for LF. It has been observed that serum exosomal miR-122 levels decrease significantly with the progression of LF, possibly due to the inhibition of miR-122, which promotes the proliferation of HSC and the expression of fibrosis-related markers (65). In metabolic-associated fatty liver disease (MAFLD), exosomal miR-27a, which is involved in the development of LF, is significantly elevated in serum and positively correlates with the degree of LF. As such, serum exosomal miR-27a has been proposed as a potential diagnostic biomarker for MAFLD-associated LF (37). The exosomal miR-574-5p expression has been found to be upregulated in LF and to positively correlate with HSC activation and collagen deposition, suggesting its potential as a biomarker for the diagnosis of LF (51). Exosomal miR-500 and miR-103-3p, which are involved in the development of LF, have also emerged as promising biomarkers for diagnosis and disease staging (49, 50). Plasma exosomal miR-155, which increases with the progression of liver fibrosis, has been proposed as a sensitive biomarker of LF. Furthermore, in patients with liver fibrosis undergoing liver transplantation, high expression of exosomal miR-155 has been associated with a worse clinical outcome compared to low expression (66).Some exosomal miRNAs have been demonstrated to be no less effective than existing diagnostic tools for diagnosing LF. Specifically, exosomal miR-92a-3p and miR-146a-5p are more advantageous in diagnosing advanced fibrosis associated with chronic hepatitis B than LSM, APRI, and FIB-4 (64). Additionally, using exosomal miR-155 in combination with Type IV collagen (CIV), hydroxyproline (Hyp), and aspartate aminotransferase (AST) may improve the diagnostic accuracy of LF (66). Exosomal miR-122 is more effective in identifying advanced liver fibrosis when combined with FIB-4 and transient elastography (65). Therefore, the combined use of exosomal miRNA with clinically used LF diagnostic methods may significantly enhance the diagnosis.

Exosomes carrying circRNA and lncRNA also have the potential to become biomarkers of LF. In patients with liver failure, serum exosomal circ-Death Inducer-Obliterator 1(circ-DIDO1) levels are decreased, and exosomal circDIDO1 has been shown to inhibit HSC activation in LF. It suggests that Serum exosomal circ-DIDO1 may serve as a diagnostic biomarker for LF (67). Xiao et al. demonstrated that serum exosomal lncRNA-H19 levels were positively correlated with the severity of LF in patients with biliary atresia. This finding suggests that exosomal lncRNA-H19 may serve as a potential biomarker for cholestatic LF in biliary atresia (68). Research has demonstrated that exosomal lncRNA-MALAT1 is involved in arsenite-induced LF and is upregulated in the sera of individuals exposed to arsenite, indicating its potential as a biomarker for arsenicosis-induced LF (43). The cluster of differentiation of 44 (CD44) can promote the progression of LF and is upregulated in serum exosomes of patients with congestive hepatopathy. This suggests that serum exosomal CD44 may have the diagnostic ability for LF in congestive hepatopathy (69).

The detection of exosomal miRNAs, circRNAs, and lncRNAs from serum or plasma is a promising approach to enhance the diagnosis of LF. These exosomal ncRNAs can be stably present in blood, and the current methods for isolation, extraction, and detection of these molecules are now becoming mature (11). Furthermore, these exosomal non-coding RNAs can be used as biomarkers to monitor the progression of LF caused by different diseases and to distinguish between the disease’s early and advanced stages. The combined use of exosomal miRNA with clinically used LF diagnostic methods may significantly enhance the diagnosis. Moreover, monitoring the progression of LF over time through the use of exosomes may help develop more effective treatment strategies. Despite the promising results of the current studies, some limitations must be addressed. Specifically, the current studies are limited in sample size and the number of biomarkers tested, and the exosome detection technology is not yet suitable for widespread clinical use. Further research is necessary to ensure the accuracy and reliability of these biomarkers in different populations and clinical settings. Also, cost-effective and efficient exosome detection methods must be developed to promote their clinical use.

### 4.2 Therapeutic potential of exosomes in LF

Despite recent improvements in LF treatment, it does not meet the current medical needs. Effective and safe anti-fibrotic drugs are still needed to delay and reverse LF and improve patient prognosis (2). In recent years, the therapeutic potential of exosomes has attracted significant attention, providing new ideas for treating LF (23, 70).

#### 4.2.1 Potential of exosomes as therapeutic agents for LF

Native exosomes, which carry biomolecules from parental cells and serve as intercellular communication carriers, have emerged as a novel therapeutic strategy for various diseases (71). Numerous studies have demonstrated the anti-fibrotic properties of native exosomes, indicating their potential as therapeutic agents for LF (**Table 2**). Mesenchymal stem cells(MSC) play a crucial role in cell therapy. They have been extensively utilized in research and clinical trials to treat multi-system diseases, such as LF. It is generally accepted that MSCs mainly exert their effects through paracrine mechanisms mediated by exosomes and other factors (82, 83). Studies have indicated that Mesenchymal stem cell-derived exosomes(MSC-EXO) possess therapeutic properties, including reducing liver inflammation and fibrosis, stimulating liver cell regeneration, and improving liver function. Tian et al. found that MSC-EXO could ameliorate LF by promoting the shift of macrophages from the M1 pro-inflammatory phenotype to the M2 anti-inflammatory phenotype. This effect is attributed to the ability of miR-148a delivered by MSC-EXO to regulate KLF6/STAT3 signaling (72). Bone marrow mesenchymal stem cell-derived exosome(BMSC-EXO) have been demonstrated to be more effective than BMSC in alleviating LF It has been reported that BMSC-EXO can inhibit HSC activation by inhibiting the Wnt/β-catenin pathway, thereby alleviating CCl4-induced LF in rats (73). Furthermore, Ma et al. found that BMSC-derived exosomal circCDK13 could regulate Milk fat globulin-egf factor 8(MFGE8) expression through miR-17-5p/KAT2B to inhibit LF (74). Human umbilical cord mesenchymal stem cell-derived exosomes (hucMSC-EXO) also could be a potential therapeutic approach for LF. HucMSC-EXO could inactivate the TGF-β1/Smad signaling pathway and inhibit epithelial-mesenchymal transition (EMT) in the liver (75). Moreover, hucMSC-EXO may reduce oxidative stress and inhibit apoptosis in LF (76). Glutathione peroxidase 4 (GPX4) is a key regulator of ferroptosis. HucMSC-EXO has been shown to significantly reduce GPX4 expression in aHSC and collagen deposition in mouse models of LF. It is attributed to Beclin 1(BECN1) delivered by hucMSC-EXO, which could induce ferroptosis in HSC by modulating the xCT/GPX4 axis and thus alleviate LF (77).

**Table 2:** Potential role of exosomes as therapeutic agents for LF.

Source of exosomes	Experimental model	Exosome - Cargos	Species	Delivery of exosomes	Treatment Mechanism	Potential therapeutic implication	References
MSC	CCl4	miR-148a	Mice	Intravenous injection	Regulate KLF6/STAT3 signaling and promote the conversion of macrophages from M1 pro-inflammatory phenotype to M2 anti-inflammatory phenotype	Reduce liver inflammation and fibrosis, improve liver function	(72)
BMSC	CCl4	Unknown	Rat	Intravenous injection	Inhibit the Wnt/β-catenin pathway to suppress HSC activation	Reduce liver collagen accumulation and inflammatory response, promote hepatocyte regeneration and improve liver function	(73)
BMSC	Thioacetamide	circCDK13	Mice	Intraperitoneal injection	regulating the expression of MFGE8 through miR-17-5p/KAT2B axis.	Reduce collagen deposition and fibrosis in the liver	(74)
hucMSC	CCl4	Unknown	Mice	Liver Injection	Inactivate the TGF-β1/Smad signaling pathway and inhibit epithelial mesenchymal transition	Alleviate liver inflammation and collagen deposition and significantly restore serum AST activity	(75)
hucMSC	CCl4	Unknown	Mice	Intravenous injection	Reduce oxidative stress and inhibit apoptosis	Relieve collagen deposition in liver	(76)
hucMSC	CCl4	BECN1	Mice	Intravenous injection	Regulate the xCT/GPX4 axis to induce ferroptosis in HSC	Alleviates collagen deposition in liver and reduces GPX4 expression in activated HSC	(77)
hESC	CCl4 and alcohol	miR-6766-3p	Mice	Intravenous injection	Targeting the TGFβRII-SMADS pathway and thereby attenuating HSC activation	Reduce hepatic collagen deposition, attenuate hepatic fibrosis, and promote liver reconstruction and liver function recovery	(78)
HepG2 cell	CCl4	miR-423-5p	Mice	Intravenous injection	Inhibit HSC differentiation	Reduce collagen deposition and fibrosis in the liver, and improve liver function	(35)
NK	CCl4	Unknown	Mice	Intravenous injection	Inhibit the activation of HSC	Reduce collagen deposition and fibrosis in the liver, and improve liver function	(79)
NK	TGF-β1	miR-223	LX-2 cell	–	Inhibit HSC activation by inhibiting autophagy	Reduce HSC proliferation and the levels of α-SMA and CoL1A1	(63)
ADSC	DEN, CCl4	Unknown	Mice	Intravenous injection	Inhibit HSC activation and remodel hepatocyte glutamine synthetase-mediated glutamine and ammonia homeostasis	Improve liver function, reduce liver collagen deposition and reverse the pro-fibrotic phenotype	(80)
UCB	CCl4	Unknown	Mice	Intravenous injection	Inhibit the TGF-β-ID1 signaling pathway to block HSC activity	Improve liver function and reduce collagen deposition	(81)

MSC, mesenchymal stem cell; BMSC, bone marrow-derived stem cells; CCl4, carbon tetrachloride; hucMSC, human umbilical cord mesenchymal stem cell; EMT, epithelial-mesenchymal transition; HSC, hepatic stellate cell; GPX4, glutathione peroxidase 4; NK, natural killer cell; DEN, diethylnitrosamine; ADSC, adipose-derived stromal cell; UCB, umbilical cord plasma.

Exosomes from other sources, such as human embryonic stem cells (hESC), are also promising therapeutic agents for LF. Wang et al. demonstrated that 3D hESC-derived exosomes (3D-hESC-EXO) could reduce hepatic collagen deposition, attenuate LF, and promote liver reconstruction and liver function recovery. The anti-fibrotic effect of 3D-hESC-EXO is attributed to the delivery of miR-6766-3p, which attenuates HSC activation and alleviates LF by targeting the TGFβRII-SMADS pathway (78). Notably, the combination of nilotinib with stem cell exosomes could exert better anti-fibrotic effects in CCl4-induced LF in rats compared to their respective use (84). Safran et al. demonstrated that hepatocyte-derived exosomes can reduce collagen deposition, fibrosis and improve liver function in a mouse model of CCl4-induced LF, which is attributed to the inhibition of HSC differentiation by miR-423-5p delivered by hepatocyte-derived exosomes (35). Natural killer cell-derived exosomes (NK-EXO) have been reported to alleviate CCl4-induced LF in mice, possibly due to the high expression of miR-223 in NK-EXO that inhibits autophagy and thus attenuates HSC activation (63, 79). Wu et al. found that intravenous adipose-derived stromal cell(ADSC)-derived exosomes(ADSC-EXO) effectively improved liver function and reduced hepatic collagen deposition in mice with LF. Notably, ADSC-EXO may reverse the pro-fibrotic phenotype *in vivo* and *in vitro*. Upon more in-depth study, they concluded that ADSC-EXO improve LF by inhibiting HSC activation and remodeling hepatocyte glutamine synthetase-mediated glutamine and ammonia homeostasis (80). In addition, umbilical cord plasma-derived exosomes (UCB-EXO) may be a promising therapy for LF. Huang et al. found that UCB-EXO also exert antifibrotic effects in a mouse model of LF. The mechanism may be that UCB-EXO inhibit the TGF-β-ID1 signaling pathway to block HSC activity (81).

Native exosomes derived from various cell sources have the potential to be utilized as therapeutic agents for LF. Combining natural exosomes with other therapeutic strategies is a promising approach to enhance the clinical efficacy of LF. Despite the potential of native exosomes as therapeutic agents, there remain numerous challenges to their successful application in clinical practice. The poor targeting property of native exosomes is a major obstacle to their successful application as therapeutic agents (85). However, engineered exosomes offer a potential solution to this issue. For instance, exosomes modified with targeted peptides of aHSC can increase the efficiency of targeted hepatic stellate cell delivery and improve efficacy in treating LF (86). The use of native exosomes as LF therapeutic agents has other drawbacks, including a lack of long-term studies to assess safety and efficacy, a lack of comprehensive exploration of therapeutic mechanisms, and the potential for prohibitive costs. Additional efforts and further research are required to facilitate the clinical application of exosomes as therapeutic agents for LF.

#### 4.2.2 Potential of exosomes as therapeutic targets for LF

Given the different roles of exosomes in the development of LF, exosomes and the cargoes they carry constitute numerous potential targets to influence the progression of LF. As mentioned above, exosomes play a key role in intercellular communication between HSC and other cells in the progression of LF. In light of several studies, we propose that affecting HSC function by targeting exosomes is a promising therapeutic direction for LF.

Targeting exosomes as a therapeutic approach to inhibit LF may be achieved by altering the cargo composition of exosomes, affecting the formation and release of exosomes, and blocking the propagation of exosomes (12). Exosomes have been implicated in the progression of liver fibrosis through the miRNA they carry. Luo et al. found that miR-27a inhibitors could reverse the promotion of HSC proliferation and activation by hepatocyte-derived exosomal miR-27a, suggesting that exosomal miR-27a is a potential therapeutic target for MAFLD-associated LF (37). Similarly, Wang et al. demonstrated that inhibition of miRNA-33 expression in Schistosoma japonicum egg-derived exosomes could reduce the extent of LF in schistosomiasis. MiRNA-33 is capable of promoting the activation of HSCs (48). Additionally, inhibition of miR-500 in macrophage-derived exosomes may also inhibit the proliferation and activation of HSC (49). Thus, exosomes can be targeted to treat LF by inhibiting the miRNAs they carry that promote disease progression. Regulation of the parent cells of exosomes can control the production and release of exosomes. Hou et al. found that IL-6 treatment could promote macrophages to release exosomes rich in antifibrotic miR-223, which could be transferred to hepatocytes to inhibit LF, providing a therapeutic target for NAFLD-associated LF treatment (87). GW4869 is a neutral sphingomyelinase inhibitor that blocks exosome production by preventing the formation of ILV (88). GW4869 could reduce miR-192 levels in HCV replicating hepatocyte-derived exosomes, a major regulator of HCV-mediated LF, thereby impeding HSC transdifferentiation and α-SMA accumulation (41). Fang et al. found that treatment of exosomes released from aHSC cells with annexin may block the activation of qHSC cells (53). It provides a new idea for blocking the spread of exosomes and inhibiting LF.

Traditional Chinese medicine(TCM) is receiving increasing attention in treating LF. Many natural compounds and extracts of herbs are considered beneficial in the treatment of LF, however, their therapeutic mechanisms are not yet clear (89). Salidroside is derived from the Chinese herb *Rhodiola rosea*. Salidroside has been reported to inhibit LF by reducing the exosomal SphK1-induced activation and migration of HSC (90). Therefore, exosomes can serve as drug targets for TCM in the treatment of LF, providing directions for further research on the therapeutic mechanism of TCM for LF.

The use of exosomes as therapeutic targets offers promising avenues for novel treatment strategies for LF, and provides novel insights into the mechanisms of anti-fibrotic drugs. However, further research is required to elucidate the details of exosome involvement in LF and its regulatory mechanisms, in order to drive the development of anti-fibrotic therapies targeting exosomes.

#### 4.2.3 Potential of exosomes as drug delivery system for LF

**Figure 3:**
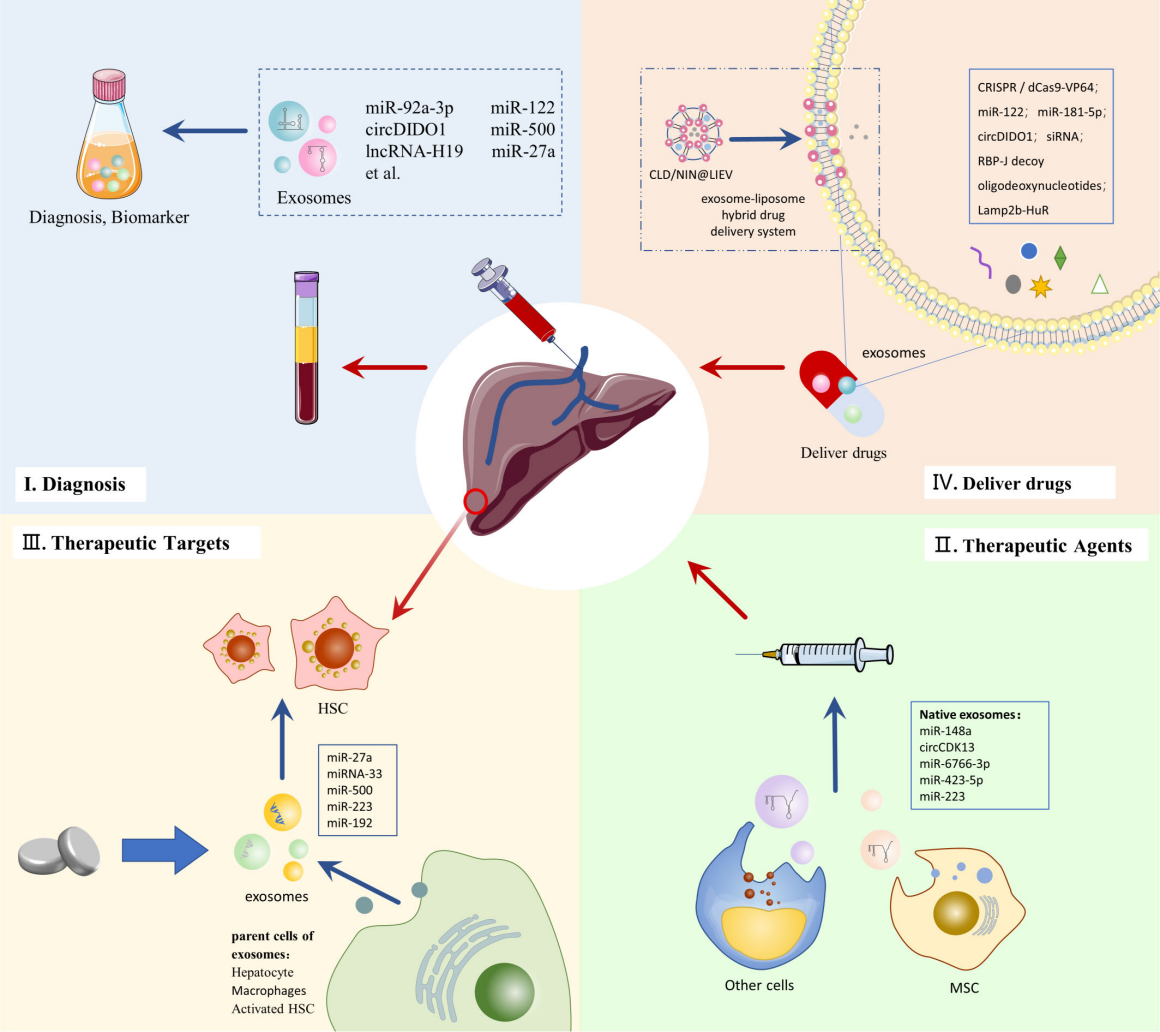
Clinical application of exosomes in LF.

Exosomes play an important role in intercellular communication and can transfer substances such as proteins, nucleic acids and lipids from donor cells to recipient cells. Exosomes have better biocompatibility and lower immunogenicity. They are able to penetrate tissues, diffuse into the bloodstream, and also cross biological barriers, such as the blood-brain barrier. In addition, exosomes have a strong ability to home in on target tissues or cells (23, 29). Therefore, exosomes have been widely used in drug delivery studies (91). Compared to other drug delivery systems (e.g. liposomes), exosome-based drug delivery systems offer advantages in terms of increased stability, reduced toxicity, and targeted delivery (92, 93). Given these advantages of exosomes, they have been noted as a promising drug delivery system for LF (**Figure 3**).

While advanced gene therapy technologies offer new therapeutic promise for LF, there are challenges, such as the need for safer and more effective delivery systems (94). Exosome-based drug delivery systems may provide the impetus for developing gene therapy for LF. Clustered regularly interspaced short palindromic repeats (CRISPR)/CRISPR-associated protein 9 (Cas9) gene editing is a promising therapeutic technology, but its low intracellular delivery efficiency hinders its clinical application (95). The CRISPR-Cas9 gene editing deliverables contain plasmids and Cas9 ribonucleoprotein (RNP) (96). Luo et al. used AML12 cell-derived exosomes to efficiently deliver CRISPR/dCas9-VP64 plasmid DNA into HSC, providing new feasibility for gene therapy of LF (97). The large size of Cas9 ribonucleoprotein (RNP) limits its delivery vector selection and clinical use in gene therapy. Wan et al. loaded Cas9 RNP into HSC-isolated exosomes by electroporation. They promoted the effective delivery of Cas9 RNP and its accumulation in liver tissues, and this delivery system plays a strong therapeutic potential in LF (96).

Using exosomes as *in vivo* carriers for nucleic acids delivery is receiving increasing attention. Due to its advantages, such as immunological and homing properties, MSC-EXO is widely used in studies to deliver therapeutic RNAs (98). MiR-122 can negatively regulate the proliferation and collagen production of HSCs (99). By packaging miR-122 into adipose-derived MSC (AMSC) secreted exosomes, miR-122 can be target delivered to HSC and exert anti-LF effects (100). Similarly, engineering AMSC-derived exosomes could also serve as delivery vehicles for miR-181-5p, a possible regulator of autophagy in HSC, and thus exert therapeutic effects on a mouse model of LF (101). In addition, MSC-EXO has demonstrated the ability to deliver circDIDO1 to HSC for antifibrotic effect, making it a promising delivery vehicle for circRNA therapy *in* vivo (67). STAT3 is an important transcription factor in the pathogenesis of LF. Tang et al. loaded STAT3-targeting antisense oligonucleotide and small interference RNA (siRNA) onto MSC-EXOs separately, and with the delivery advantage of exosomes, these two approaches effectively suppressed STAT3 levels and ameliorated LF in LF mice (102). Osteopontin plays an important role in the pathogenesis of LF. Tang et al. used electroporation to enable exosomes to carry siRNA targeting osteopontin to inhibit LF. This exosome delivery system showed high uptake and low toxicity, effectively improving the low delivery efficiency of siRNA to target organs (103). In addition, He et al. found that intravenous injection of exosomes loaded with the transcription factor RBP-J decoy oligodeoxynucleotides effectively inhibited Notch signaling in macrophages and ameliorated LF in mice (104).

The advantages of exosome-targeted delivery may also contribute to therapies that interfere with RNA function *in vivo*. Li et al. use exosomes to deliver engineered RNA-binding proteins for targeting and degrading specific RNAs in lysosomes. They found that delivery of acidified exosomes engineered with Lamp2b-HuR attenuated LF while reducing some inflammatory genes (105). In addition, drug delivery systems that hybridize exosomes with other nanoparticles offer new strategies for treating LF. In response to the limitations of LF treatment due to inefficient drug delivery and Kupffer cell-induced inflammation, Ji et al. developed an exosome-liposome hybrid drug delivery system (LIEV) to load clodronate(CLD) and nintedanib (NIN). This drug delivery system enhances the inhibitory effect of CLD on Kupffer cells. It increases the effective delivery of NIN to hepatic fibroblasts, providing a safer and more effective treatment for LF (106).

Although exosomes have promising applications as drug delivery vehicles for LF, the limitations of exosomes compared to other advanced drug delivery systems have hindered their clinical application. First, the extraction and isolation of exosomes can be more inefficient due to the complexity of the technology. Low yields and high costs prevent exosomes from being used as drug delivery vehicles on a large scale (107, 108). Second, exosomes are limited in their drug loading. The natural components that exosomes inherently carry limit the loading of exogenous substances (92). In addition, there are difficulties in controlling and evaluating exosome-delivered drugs. Exosomes have a high degree of heterogeneity, increasing their quality control challenge. The lack of knowledge about exosome properties and physiopathology makes it difficult to assess drug delivery efficacy (23, 107). Therefore, significant efforts addressing these challenges are necessary to facilitate the clinical application of exosome-based drug delivery systems.

## 5 Conclusion

As the vital mediator of intercellular communication, exosomes have been shown to play an essential role in the pathogenesis of LF. Exosomes primarily regulate HSCs, which have a major role in LF. Exosomes can promote LF by participating in the activation of HSCs and the interaction of HSCs with immune cells. Many serum-derived exosomes have shown great diagnostic potential in LF. In particular, several of these exosomal miRNAs are promising diagnostic and prognostic biomarkers for LF caused by different diseases. Exosomes from multiple sources, mainly MSC, have demonstrated strong anti-fibrotic potential in experimental animal models. These exosomes could be promising candidates for LF treatment agents. Several studies have elucidated the mechanisms of exosome regulating LF, providing multiple potential therapeutic targets for LF. Affecting the function of HSCs by targeting exosomes may be a new idea in the development of anti-LF therapies. In addition, exosomes have the potential to serve as a delivery system for LF drugs. Engineered exosomes can improve the delivery efficiency of various anti-LF agents such as non-coding RNAs *in vivo*, while providing new feasibility for various LF therapies such as gene therapy.

Although exosomes have significant diagnostic and therapeutic potential in LF, there is still a long way to go before they are available for clinical use. Technical issues such as how to efficiently extract and detect exosomes are obstacles to the translation of existing research results to the clinic (109). Despite these difficulties, further exploration of the mechanisms of exosomes and the development of exosome-based diagnostic and therapeutic approaches are needed to benefit patients with LF.

## Author contributions

YL and KL searched the literature and wrote the first draft of the manuscript. YZ and JW drew the figures. YY processed the tables in the manuscript. CZ and PG contributed to the revision of the manuscript. All authors contributed to the article and approved the submitted version.
